# Supplementary material for: Clonal Hematopoiesis and Outcomes After High-Dose Chemotherapy and Autologous Stem Cell Transplantation in Patients with AML, Myeloma, and Lymphoma
Source: Int J Mol Sci. 2025 Aug 19;26(16):8021. doi: 10.3390/ijms26168021 (PMC12386481; doi:10.3390/ijms26168021)
Supplement: Supplementary file 1 [file ijms-26-08021-s001.zip › ijms-3762385-supplementary.pdf]

Supplementary Materials:

Clonal hematopoiesis and outcomes after high-dose chemotherapy and autologous stem cell transplantation in patients with AML, myeloma and lymphoma

Corinne Natalie Schmid [1], Katharina Sponagel [1], Ulrike Bacher [2], Katja Seipel [3], Naomi Porret [2], Gertrud Wiedemann [2], Michèle Hoffmann [1], Michael Daskalakis [2], Thomas Pabst [1*]

Supplementary Table S1: CH associated somatic gene mutations

Disease	Patient	Gene	Mutation(s)	VAF (%)	2 genes mutated
AML	1	*ASXL1*	p.Glu635Argfs*15	42	yes
AML	2	*ASXL1*	p.Val818Metfs*4	49	no
AML	10	*DNMT3A*	p.Ala226Trpfs*89	8	no
AML	7	*DNMT3A*	p.Arg635Profs*7	3.1	yes
AML	3	*DNMT3A*	p.Arg882Cys	10	no
AML	5	*DNMT3A*	p.Arg882Cys	27	no
AML	6	*DNMT3A*	p.Arg882Cys	47	yes
AML	8	*DNMT3A*	p.Arg882Cys	20	no
AML	4	*DNMT3A*	p.Arg882His	27	no
AML	11	*DNMT3A*	p.Arg882His	43	no
AML	9	*DNMT3A*	p.Met801Ile	2.1	no
AML	1	*TET2*	Gly1152Valfs*5, Leu1360Trpfs*3	40, 46	yes
AML	13	*TET2*	p.Arg1261His, p.His1904Arg	11, 10	no
AML	7	*TET2*	p.Asn275Ilefs*18	1	yes
AML	12	*TET2*	p.Cys332Metfs*8, p.Lys1491Argfs*80	4, 10	no
AML	6	*TET2*	p.Leu1101Glnfs*2	43	yes
Lymphoma	16	*DNMT3A*	c.2322+1G>A	41	yes
Lymphoma	17	*DNMT3A*	p.Arg736His	3.4	no
Lymphoma	18	*DNMT3A*	p.Arg882Cys	48	yes
Lymphoma	15	*DNMT3A*	p.Val296Met	16	yes
Lymphoma	20	*TET2*	p.Arg1261Gly	43	no
Lymphoma	15	*TET2*	p.Leu1819*	20	yes
Lymphoma	19	*TET2*	p.Pro818Leufs*6, p.Ser1611Phefs*2	46, 51	no
Lymphoma	18	*TET2*	p.Thr1122Glyfs*9	49	yes
Lymphoma	16	*TET2*	p.Tyr1628*	9	yes
Lymphoma	21	*TP53*	p.Ala161Thr	52	no
Lymphoma	22	*TP53*	p.Leu194His	11	no
Lymphoma	23	*TP53*	p.Val157Gly	10	no
Myeloma	25	*DNMT3A*	c.1851+2T>G	4.4	no
Myeloma	26	*DNMT3A*	c.2082+1G>A	8	no
Myeloma	24	*DNMT3A*	p.Arg736Cys	3.4	no
Myeloma	28	*DNMT3A*	p.Arg792Profs*6	6	no
Myeloma	31	*DNMT3A*	p.Arg882Cys	9	no
Myeloma	27	*DNMT3A*	p.Gln527*, p.Arg635Pro	6, 6	no
Myeloma	32	*DNMT3A*	p.Leu773Arg	15	no
Myeloma	30	*DNMT3A*	p.Ser689Alafs*16	2.6	no
Myeloma	29	*DNMT3A*	p.Trp795*	19	no
Myeloma	35	*TET2*	p.Ala241Val	54	no
Myeloma	34	*TET2*	p.Glu1436Gln	50	no
Myeloma	33	*TET2*	p.L1212S, p.Q1834*	14, 14	no
Myeloma	36	*TET2*	p.Thr1086Lysfs*14	44	no

Supplementary Table S2: CH associated gene mutations in myeloma, lymphoma and AML

Parameter		Myeloma (n=13)	Lymphoma (n=9)	AML (n=14)	P-value
**Mutations, n (median VAF, %)**					
	ASXL1	0	0	2 (46)	0.19
	DNMT3A	9 (7)	4 (29)	9 (20)	0.48
	JAK2	0	0	0	-
	TET2	4 (50)	5 (32)	5 (22)	0.48
	TP53	0	3 (11)	0	**0.0074**
**Mutations in two genes, n (%)**		0 (0)	3 (33)	4 (29)	0.0825
**Two mutations in the same gene, n (%)**		2 (15)	1 (11)	3 (21)	0.80

1 One patient with AML carried in parallel CH with *SRSF2*- (VAF 46%) and *IDH2*-mutation (VAF 32%)
P-value for difference between myeloma, lymphoma and AML group; VAF: variant allele frequency

Supplementary Table S3: Characteristics of HDCT/ASCT in myeloma, lymphoma and AML

Parameter		CH (n=36)	noCH (n=106)	P-value
**Duration of diagnosis until ASCT, months, median (range)**		4.4 (3-187)	5.0 (3-179)	0.29
**second HDCT/ASCT in case of relapse, n (%)**		5 (14)	22 (21)	0.46
**HDCT in myeloma, n (%)**				
	Treosulfan/melphalan	11 (85)	42 (82)	>0.99
	Melphalan	1 (8)	7 (14)	>0.99
	Treosulfan/melphalan, mabthera	0 (0)	1 (2)	>0.99
	Cytarabin/treosulfan	0 (0)	1 (2)	>0.99
	Bendamustin/melphalan	1 (8)	0 (0)	0.20
**HDCT in lymphoma, n (%)**				
	BeEAM	2 (22)	17 (45)	0.28
	BrBeEAM	2 (22)	7 (18)	>0.99
	2BeEAM	0 (0)	7 (18)	0.32
	BEAM	1 (11)	2 (5)	0.48
	POLA-BeEAM	1 (11)	1 (3)	0.35
	R-BEAM	2 (22)	1 (3)	0.0895
	Carmustin/thiotepa	1 (11)	3 (8)	>0.99
**HDCT in AML, n (%)**				
	Treosulfan/melphalan	9 (64)	11 (65)	>0.99
	Busulfan/endoxane	2 (14)	6 (35)	0.24
	Busulfan/melphalan	3 (21)	0 (0)	0.0810

CH: Clonal Hematopoiesis of indeterminate potential; noCH: patients without CH; P-value for difference between CH and noCH group; ASCT: autologous stem cell transplantation; HDCT: high dose chemotherapy; BeEAM: Bendamustine, Etoposide, Ara-C, Melphalan; BrBeEAM: Brentuximab, Bendamustine, Etoposide, Ara-C, Melphalan; 2BeEAM: Bortezomib, Bendamustine, Etoposide, Ara-C, Melphalan; BEAM: BCNU, Etoposide, Ara-C, Melphalan; POLA-BeEAM: Polatuzumab, Bendamustine, Etoposide, Ara-C, Melphalan; R-BEAM: Rituximab, BCNU, Etoposide, Ara-C, Melphalan

Supplementary Table S4: Advers events during/after ASCT

Parameter		CH (n=36)	noCH (n=106)	P-value
**Number of days in aplasia after ASCT, median (range)**		11 (9-19)	11 (8-26)	0.76
**Complications, n (%)***				
	Colitis	28 (78)	84 (79)	0.82
	Catheter-associated infection	5 (14)	22 (21)	0.46
	Pneumonia	4 (11)	15 (14)	0.78
	Sepsis	5 (14)	7 (7)	0.18
	Septic shock	1 (3)	5 (5)	>0.99
	Kidney failure	8 (22)	18 (17)	0.46
	Cardiac decompensation	5 (14)	5 (5)	0.12
	Respiratory failure	2 (6)	4 (4)	0.64
	Drug eruption / exanthema	6 (17)	9 (8)	0.21
	Atrial fibrillation	2 (6)	5 (5)	>0.99
	Esophagitis	1 (3)	4 (4)	>0.99
	Delayed hematological regeneration	4 (11)	0 (0)	**0.004**
	Liver disease	4 (11)	5 (5)	0.23
	Engraftment syndrome	2 (6)	4 (4)	0.64
	Thrombosis	0 (0)	4 (4)	0.57
	Graft failure	0 (0)	1 (1)	>0.99
	Pulmonary aspergillosis	0 (0)	3 (3)	0.57
**Microbiologic agent, n (%)***				
	E. coli	7 (19)	22 (21)	>0.99
	Clostridioides difficile	4 (11)	7 (7)	0.47
	Coagulase-negative staphylococci (CoNS)	9 (25)	28 (26)	>0.99
	Streptococcus viridans	4 (11)	8 (8)	0.50
	E. faecium	2 (6)	13 (12)	0.35
	K. pneumoniae	1 (3)	6 (6)	0.68
	Pseudomonas aeruginosa	0 (0)	4 (4)	0.57
	Aspergillus	1 (3)	6 (6)	0.68
	Viruses	4 (11)	10 (9)	0.75
	Other fungal infections	3 (8)	3 (3)	0.17
	Other bacteria	6 (17)	13 (12)	0.57
	No agent detected	13 (36)	39 (37)	>0.99

* >1 per patient possible, list not exhaustive

CH: Clonal Hematopoiesis of indeterminate potential; noCH: patients without CH; P-value for difference between CH and noCH group; ASCT: autologous stem cell transplantation

Supplementary Table S5: Clinical outcomes in myeloma patients after HDCT/ASCT

Parameter		CH (n=13)	noCH (n=51)	P-value
**Remission after ASCT, n (%)**				
	CR (complete remission)	12 (92)	37 (73)	0.27
	VGPR (very good partial remission)	0 (0)	4 (8)	0.57
	PR (partial remission)	1 (8)	6 (12)	>0.99
	SD (stable disease)	0 (0)	1 (2)	>0.99
	PD (progressive disease)	0 (0)	1 (2)	>0.99
**Relapse/progression after ASCT, n (%)**		1 (8)	6 (12)	>0.99
	Months until relapse/progression after ASCT, median (range)	72.7 (-)	8.7 (1.9-13.3)	-
**Secondary malignancy, n (%)**		1 (8)	1 (2)	0.37
	Months until second. malignancy after ASCT, median (range)	25.5 (-)	12.0 (-)	-
**Death, n (%)**		0 (0)	5 (10)	0.57
	Months until death after ASCT, median (range)	-	6.4 (0.3-12.4)	-
	Death related to the underlying disease or therapy, n (%)	-	5 (100)	-
**Follow up, months, median (range)**		13.1 (9.2-79.1)	15.2 (0.3-22.8)	0.96

CH: Clonal Hematopoiesis of indeterminate potential; noCH: patients without CH; P-value for difference between CH and noCH group; ASCT: autologous stem cell transplantation; CR: complete remission; VGPR: very good partial remission; PR: partial remission; SD: stable disease; PD: progressive disease

Supplementary Table S6: Clinical outcomes in AML patients after HDCT/ASCT

Parameter		CH (n=14)	noCH (n=17)	P-value
**Remission after ASCT, n (%)**				
	CR (Complete Remission)	14 (100)	17 (100)	>0.99
	PR (Partial Remission)	0 (0)	0 (0)	>0.99
	PD (Progressive Disease)	0 (0)	0 (0)	>0.99
**Relapse/progression after ASCT, n (%)**		6 (43)	5 (29)	0.48
	Months until relapse/progression after ASCT, median (range)	5.2 (2.2-7.2)	5.8 (2.0-13.2)	0.79
**Secondary malignancy, n (%)**		0 (0)	2 (12)	0.49
	Months until second. malignancy after ASCT, median (range)	-	165.2 (104.3-226.1)	-
**Death, n (%)**		4 (29)	2 (12)	0.37
	Months until death after ASCT, median (range)	6.9 (4.2-7.6)	17.6 (13.0-22.3)	0.13
	Death related to the underlying disease or therapy, n (%)	4 (100)	2 (100)	>0.99
**Follow up, months, median (range)**		17.8 (4.2-220.7)	24.6 (10.3-248.4)	0.15

CH: Clonal Hematopoiesis of indeterminate potential; noCH: patients without CH; P-value for difference between CH and noCH group; ASCT: autologous stem cell transplantation; CR: complete remission; PR: partial remission; PD: progressive disease
